# *N*-Amino-l-Proline Methyl Ester from an Australian Fish Gut-Derived Fungus: Challenging the Distinction between Natural Product and Artifact

**DOI:** 10.3390/md19030151

**Published:** 2021-03-12

**Authors:** Osama G. Mohamed, Zeinab G. Khalil, Robert J. Capon

**Affiliations:** 1 Institute for Molecular Bioscience, The University of Queensland, St Lucia, QLD 4072, Australia; Osama.mohamed@pharma.cu.edu.eg (O.G.M.); z.khalil@uq.edu.au (Z.G.K.); 2 Natural Products Discovery Core, Life Sciences Institute, University of Michigan, Ann Arbor, MI 48109, USA

**Keywords:** HPLC analysis, fungal metabolites, *N*-amino-l-proline methyl ester, prolinimines, 5-hydroxymethyfurfural, 2,5-furandicarboxaldehyde, Schiff base, artifact, media component

## Abstract

Further investigation into a fish gut-derived fungus *Evlachovaea* sp. CMB-F563, previously reported to produce the unprecedented Schiff base prolinimines A–B (**1**–**2**), revealed a new cryptic natural product, *N*-amino-l-proline methyl ester (**5**)—only the second reported natural occurrence of an *N*-amino-proline, and the first from a microbial source. To enable these investigations, we developed a highly sensitive analytical derivitization methodology, using 2,4-dinitrobenzaldehyde (2,4-DNB) to cause a rapid in situ transformation of **5** to the Schiff base **9**, with the latter more readily detectable by UHPLC-DAD (400 nm) and HPLC-MS analyses. Moreover, we demonstrate that during cultivation **5** is retained in fungal mycelia, and it is only when solvent extraction disrupts mycelia that **5** is released to come in contact with the furans **7**–**8** (which are themselves produced by thermal transformation of carbohydrates during media autoclaving prior to fungal inoculation). Significantly, on contact, **5** undergoes a spontaneous condensation with **7**–**8** to yield the Schiff base prolinimines **1**–**2**, respectively. Observations made during this study prompted us to reflect on what it is to be a natural product (i.e., **5**), versus an artifact (i.e., **1**–**2**), versus a media component (i.e., **7**–**8**).

## 1. Introduction

**Figure 1:**
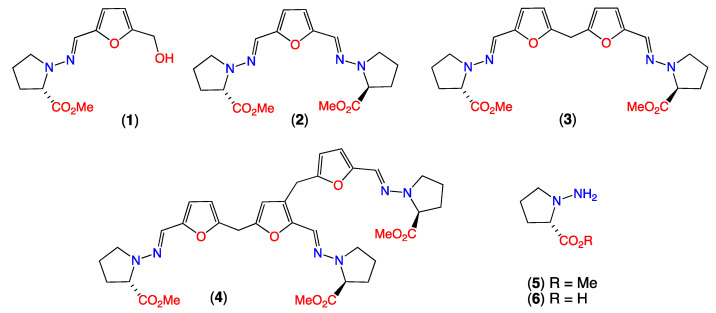
Prolinimines A–D (**1**–**4**) and *N*-amino-l-prolines **5**–**6**.

A recent 2020 review used case studies in marine natural products chemistry to illustrate the heightened reactivity of selected natural products to environmental stimuli (i.e., pH, temperature, light, oxygen, metal ions, and solvents), where isolation, handling and/or storage promoted the formation of artifacts [1]. This review concluded, for a chemical to be viewed as a natural product it was not merely enough that it be isolated from a natural source, the compound should also be detected in the fresh solvent extract prior to fractionation. One of the case studies used to illustrate this concept was that of the Schiff base prolinimines A–D (**1**–**4**) (Figure 1). The prolinimines were first reported by our lab in 2018 from a fish gastrointestinal tract-derived fungus *Trichoderma* sp. CMB-F563 (herein reclassified as *Evlachovaea* sp. CMB-F563) [2]. At that time, it was noted that while standard isolation and handling techniques applied to CMB-F563 rice grain media cultivations yielded prolinimines B–D (**2**–**4**), high performance liquid chromatography (HPLC) analysis of fresh EtOAc extracts prior to any fractionation detected only prolinimines A–B (**1**–**2**). Collectively, these observations prompted us to designate **1**–**2** as natural products, and **3**–**4** as artifacts. Consistent with this, we went on to document an in situ acid-mediated mechanism for the transformation of **1** to **3**–**4**, informing a successful convergent biomimetic synthesis. 

Intrigued by the structures and reactivity of the prolinimines, this latest report provides an account of our continued investigations into CMB-F563, leading to discovery of the new, highly cryptic natural product, *N*-amino-l-proline methyl ester (**5**). Our discovery of **5** prompted us to question the status of prolinimines A–B (**1**–**2**), and to reflect on how we might better classify chemicals isolated from microbial cultures as natural products, artifacts and/or media constituents.

## 2. Results

### 2.1. Uninoculated Rice Media Is the Source of Furan Aldehydes

**Figure 2:**
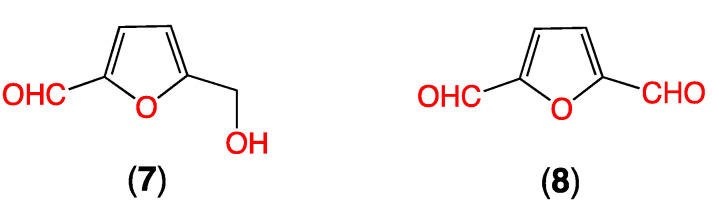
Furan aldehyde culture media components.

Based on our knowledge of prolinimines A–B (**1**–**2**) [2], we speculated the prolinyl residues were biosynthetically derived from *N*-amino-l-proline methyl ester (**5**) or its carboxylic acid **6** (Figure 1), while the furanyl residues were derived from 5-hydroxymethyfurfural (**7**) in the case of **1**, and 2,5-furandicarboxaldehyde (**8**) in the case of **2** (Figure 2). Significantly, it is well-known that the furans **7** and **8** are generated by thermal transformation of monosaccharides (i.e., fructose, glucose) and disaccharides (i.e., saccharose, lactose) during the preservation and/or preparation of carbohydrate containing foods, including dried fruits, fruit juices and jams, honeys, coffees, breads, infant dairy products, breakfast cereals, biscuits, and rice wine [3,4,5,6]. Drawing on this knowledge, it seemed likely that **7** and **8** were formed during autoclaving of rice grain media prior to fungal inoculation and cultivation.

**Figure 3:**
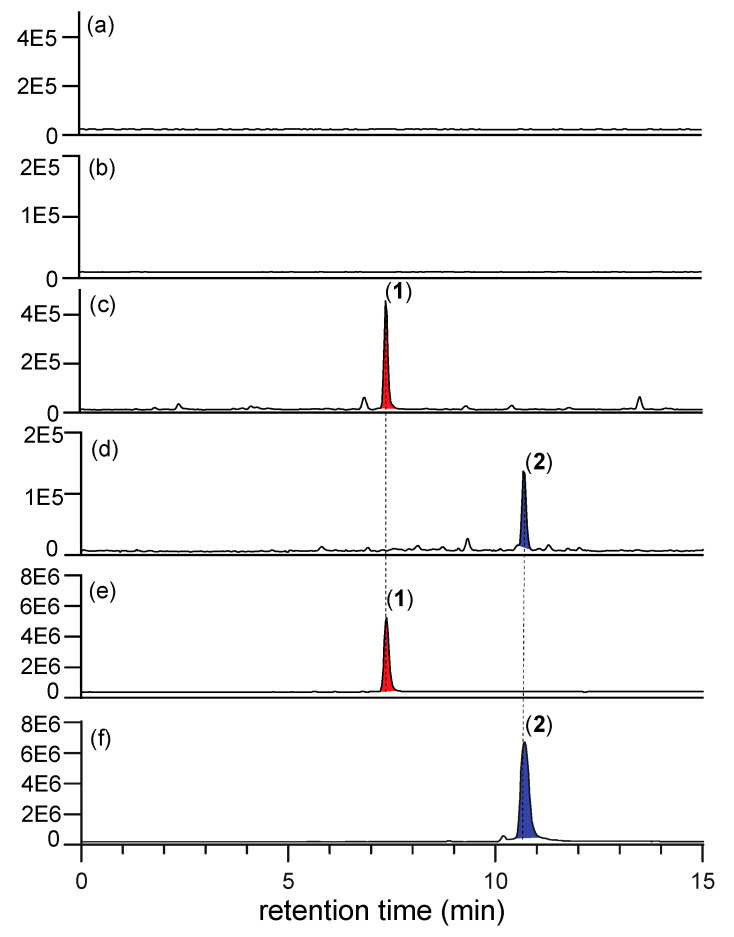
HPLC-ESI-MS-SIE traces of EtOAc extracts from uninoculated (autoclaved) rice grain media (**a**,**b**) without, and (**c**,**d**) with addition of *N*-amino-l-proline methyl ester (**5**), to reveal (**a**) M + H, *m*/*z* 253, **1** not detected, (**b**) M + H, *m*/*z* 377, **2** not detected, (**c**) M + H, *m*/*z* 253, **1** detected, and (**d**) M + H, *m*/*z* 377, **2** detected; with comparison to authentic standards of (**e**) **1** and (**d**) **2**; confirming that the furans **7** and **8** are present in uninoculated, autoclaved media, and that they react spontaneously with exogenous **5** to form prolinimines **1**–**2**.

To test this hypothesis, samples of autoclaved (but uninoculated) rice grain media were extracted with EtOAc, with and without coaddition of authentic samples of *N*-amino-l-proline methyl ester (**5**). Aliquots taken from each extract at regular intervals over 24 h were subjected to HPLC analysis with electrospray mass spectrometric detection with single ion extraction (HPLC-ESI-MS-SIE). Prolinimines A (**1**) and B (**2**) were only detected in rice grain media treated with **5**, confirming the presence of the furan precursors **7**–**8** and the absence of endogenous **5** in uninoculated rice grain media, and the ability of **7**–**8** and **5** to undergo a facile nonenzyme mediated reaction in EtOAc to form the Schiff bases **1**–**2** (Figure 3).

### 2.2. Detection of N-Amino-l-Proline Methyl Ester (**5**) and Its Free Carboxylic Acid 6 Using 2,4-Dinitrobenzaldehyde (2,4-DNB) as an In Situ Derivatizing Agent

**Figure 4:**
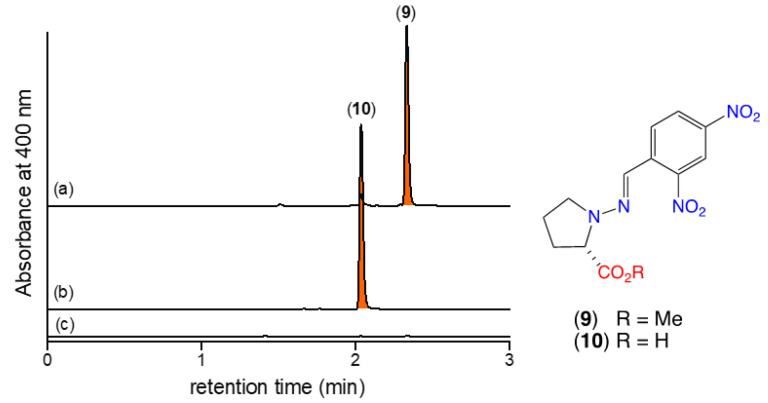
UHPLC-DAD (400 nm) traces of EtOAc extracts of uninoculated M1 broth treated with (**a**) **5** and 2,4-DNB to reveal the Schiff base **9**, (**b**) **6** and 2,4-DNB to reveal the Schiff base **10**, (**c**) 2,4-DNB to reveal no Schiff bases; confirming that **5** and **6** are not present in uninoculated M1 broth media, but that under these conditions they will react spontaneously with 2,4-DNB to form the readily detectable Schiff bases **9** and **10**, respectively.

As the *N*-amino-l-prolines **5** and **6** are polar, water soluble, low molecular weight compounds with negligible UV-vis chromophores, they are particularly difficult to detect in fungal cultivation extracts. To overcome this challenge, we developed a sensitive analytical procedure based on in situ chemical derivatization. Synthetic samples of **5** and **6** in M1 liquid broth media were treated with 2,4-dinitrobenzaldehyde (2,4-DNB), effecting rapid and quantitative in situ transformation to the Schiff bases **9** and **10**, respectively. (Note—the need to have a liquid phase necessitated using M1 broth rather than rice grain media). We specifically chose 2,4-DNB as the derivatizing agent due its strong UV chromophore and the presence of the electron withdrawing nitro groups in *ortho* and *para* positions, which prime the carbonyl group to nucleophilic attack and enhance imine/hydrazone stability. Importantly, the Schiff bases **9** and **10** proved far more amenable to UHPLC-DAD analysis than underivatized **5** and **6** (Figure 4). These studies confirmed that neither **5** or **6** were present in uninoculated M1 broth, but that if added, their presence could be revealed by in situ derivatization with 2,4-DNB as the Schiff bases **9** and **10**.

### 2.3. N-Amino-l-Proline Methyl Ester (**5**) (but Not the Free Carboxylic Acid 6) in CMB-F563 Rice Grain Media Cultivations

**Figure 5:**
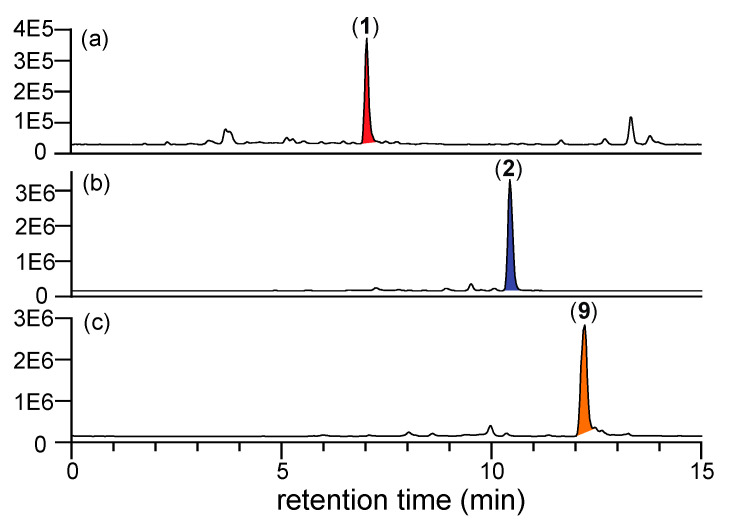
HPLC-ESI-MS-SIE traces of rice grain media cultivations of CMB-F563 extracted with EtOAc treated with 2,4-DNB, revealing (**a**) M + H, *m*/*z*, 253, **1**, (**b**) M + H *m*/*z* 377, **2** and (**c**) M + H, *m/z* 323, **9**; confirming the presence of **5** but not **6** in EtOAc extracts of rice grain media cultivations of CMB-F563.

With authentic standards of **5**–**6** and **9**–**10** in hand, as well as a sensitive analytical method for detecting **9**–**10**, 30 d CMB-F563 rice grain cultivations were extracted with EtOAc, with and without coaddition of 2,4-DNB. Aliquots taken from each EtOAc extract at regular intervals over 48 h were subjected to HPLC-DAD-ESI-MS-SIE analysis, to detect (as expected) prolinimines A–B (**1**–**2**) in all samples. More significantly, the Schiff base **9** was detected in extracts exposed to 2,4-DNB, confirming the presence of **5** in the crude EtOAc extract, whereas failure to detect the Schiff base **10** indicated the absence of the free acid **6** from all extracts (Figure 5). From this study we can confirm that **5** and prolinimines **1**–**2** are present in the EtOAc extract of CMB-F563 rice grain media cultivations.

### 2.4. Furans **7**–**8** in Uninoculated M1 Broth Media

Having established that (i) uninoculated but autoclaved rice grain media is a source of the furans **7**–**8**, (ii) CMB-F563 rice grain media cultivations are a source of the aminoproline **5**, and that (iii) **7**–**8** react rapidly with **5** in situ (i.e., in media or EtOAc) to generate the prolinimines **1**–**2**, we were keen to better understand the nature of the “biosynthetic/chemical” relationship between **1**–**2** and the precursors **5** and **7**–**8**. To achieve this we, counterintuitively, turned our attention to CMB-F563 M1 broth cultivations which do not produce **1**–**2**. We speculated that as M1 broth media possessed far lower levels of carbohydrates than rice grain media, during autoclaving M1 broth would not produce significant levels of the key furan precursors **7**–**8**. To confirm this hypothesis, uninoculated (autoclaved) M1 broth media was extracted with EtOAc, with and without coaddition of the aminoproline **5**. Subsequent UHPLC-DAD analysis failed to detect prolinimines **1**–**2** in any extract, confirming that uninoculated M1 broth media does not contain significant levels of the furans **7**–**8**.

### 2.5. N-Amino-l-Proline Methyl Ester (**5**) in CMB-F563 M1 Broth Media Cultivations

**Figure 6:**
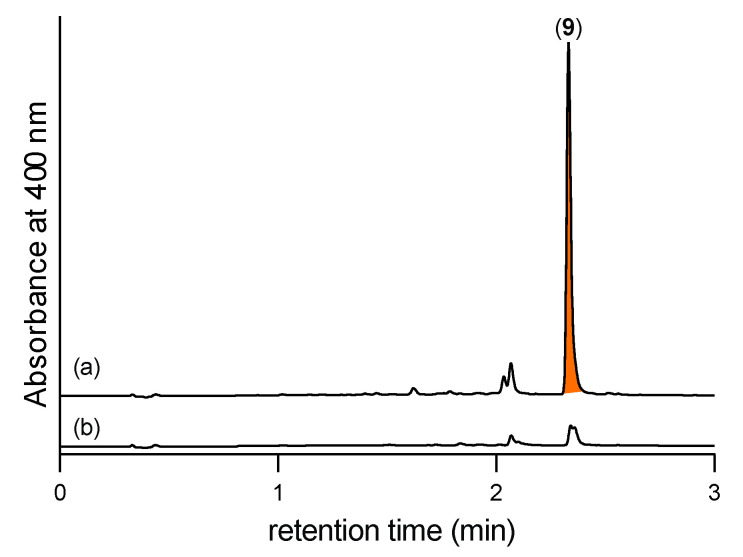
UHPLC-DAD (400 nm) traces of M1 broth media cultivations of CMB-F563 extracted with EtOAc (**a**) with and (**b**) without coaddition of 2,4-DNB; confirming that M1 broth media cultivations of CMB-F563 produce **5** (revealed as the Schiff base **9**).

Next, we hypothesized that M1 broth media cultivations of CMB-F563 produced **5**, which, due to its cryptic properties went undetected. To confirm this hypothesis, M1 broth media cultivations were extracted with EtOAc, with and without coaddition of 2,4-DNB. Unsurprisingly, UHPLC-DAD analysis failed to detect **1**–**2** in any extract, however, extracts supplemented with 2,4-DNB contained the Schiff base **9**, confirming that M1 broth cultivations of CMB-F563 did produce *N*-amino-l-proline methyl ester (**5**) (Figure 6).

### 2.6. To Secrete or Not Secrete N-Amino-l-Proline Methyl Ester (5)

**Figure 7:**
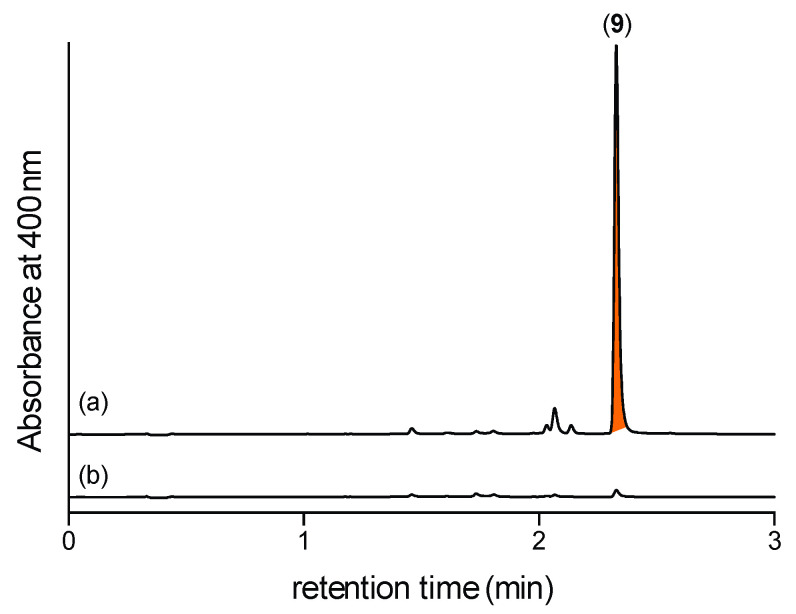
UHPLC-DAD (400 nm) traces of M1 broth cultivations of CMB-F563 partitioned into (**a**) mycelia and (**b**) supernatant, extracted with EtOAc supplemented with 2,4-DNB confirming that **5** is retained in (i.e., not secreted from) fungal mycelia.

**Figure 8:**
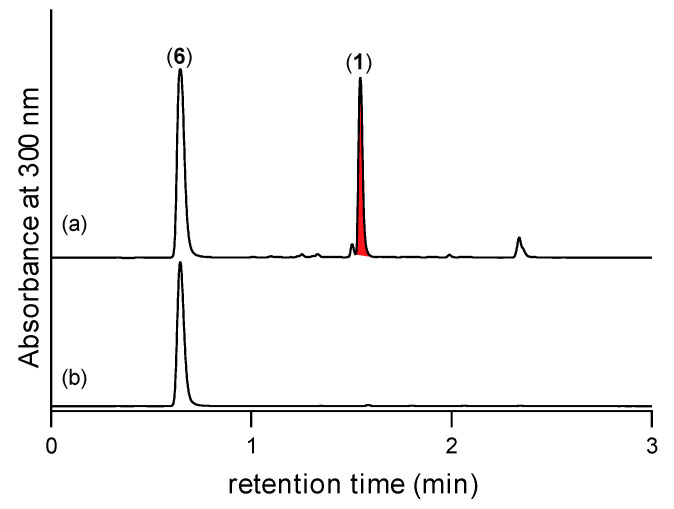
UHPLC-DAD (300 nm) traces of M1 broth cultivations of CMB-F563 partitioned into (**a**) mycelia and (**b**) supernatant, extracted with EtOAc supplemented with furan **7** confirming that **5** is retained in (i.e., not secreted from) fungal mycelia.

Next, we addressed the question of whether **5** was retained within or secreted from CMB-F563 mycelia during M1 broth media cultivation. This was achieved by careful separation of intact CMB-F563 mycelia from the cultivation broth (a task not possible for rice grain cultivations), followed by separate extraction of the mycelia and supernatant broth with EtOAc containing 2,4-DNB. Significantly, UHPLC-DAD analysis detected the Schiff base **9** in the mycelia but not supernatant extract, confirming that under these cultivation conditions **5** was retained within the fungal mycelia (Figure 7). Based on the above we propose that during EtOAc extraction, CMB-F563 mycelia rupture and allow **5** to come in contact and rapidly react with 2,4-DNB to form the Schiff base **9**. Finally, we repeated this experiment with EtOAc supplemented with the furan **7**, to detect **1** in the mycelia but not supernatant extract (Figure 8). These studies demonstrate that CMB-F563 M1 broth media cultivations can be made to produce prolinimines **1**–**2**, but only when the broth media is supplemented with the furans **7**–**8**. This raises an interesting dilemma. As prolinimines **1**–**2** were not produced by CMB-F563, but were instead formed during solvent extraction of CMB-F563 cultivations, should **1**–**2** be viewed as natural products or artifacts?

### 2.7. N-Amino-l-Proline Methyl Ester (**5**) in Other CMB-F563 Broth Media Cultivations

**Figure 9:**
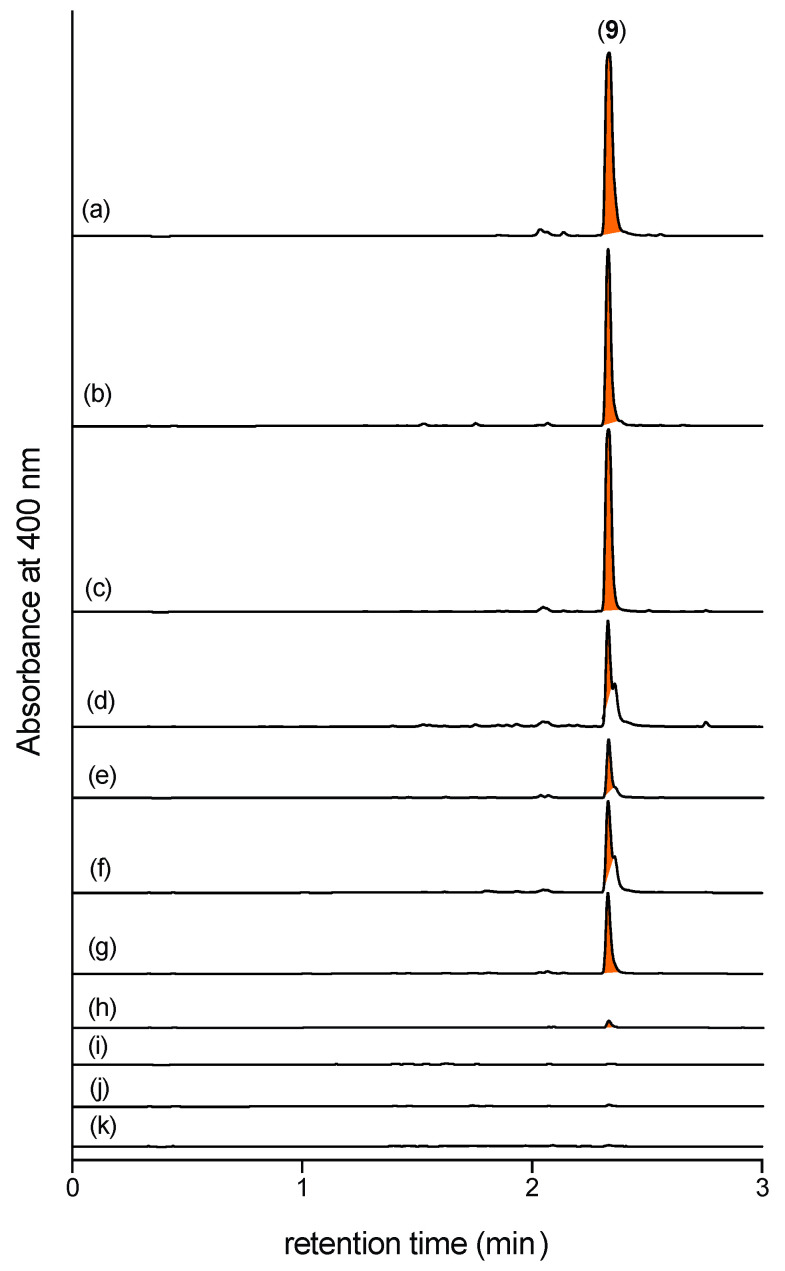
UHPLC-DAD (400 nm) traces of EtOAc (+ 2,4-DNB) extracts prepared from CMB-F563 cultures in (**a**) PYG, (**b**) ISP2, (**c**) TSB (high levels of **9**); (**d**)YES, (**e**) M1, (**f**) M2, and (**g**) PY (significant levels of **9**); (**h**) Czapek, (**i**) SDB, (**j**) basal, and (**k**) PDB (no detectable **9**): confirming that CMB-F563 produces the cryptic natural product **5** under a range of different broth media.

Having established *N*-amino-l-proline methyl ester (**5**) as a cryptic natural product, and armed with a sensitive analytical method capable of detecting even trace levels in extracts, we investigated the ability of CMB-F563 to produce **5** in different broth media. A panel of 11 CMB-F563 broth media cultivations (MATRIX) [7] were extracted with EtOAc treated with 2,4-DNB, to reveal high levels of production of the Schiff base **9** (and by inference **5**) in peptone yeast glucose (PYG), ISP2 and tryptone soy (TSB), significant levels in M1, M2, peptone yeast (PY), and yeast extract sucrose (YES), and no production in sabouraud dextrose (SDB), potato dextrose (PDB), Czabek, and basal (Figure 9).

### 2.8. N-Amino-l-Proline Methyl Ester (**5**) in Broth Media Cultivations of Other Fungi

We next set out to determine if **5** was produced by fungi other than CMB-F563. Although we tested PYG broth media cultivations for a range of marine-derived fungi; including *Fusarium* sp. CMB-NF041, *Aspergillus terreus* CMB-M0231F (dihydroisoflavipucine), *Penicillium roseopurpureum* CMB-MF038 (roseopurpurins) [8], *Eupenicillium javanicum* CMB-MF036, *Chaunopycnis* sp. CMB-MF028 (chaunopyrans, chaunolidones, chaunolidines) [9], *Beauvaria bassiana* CMB-MF026, *Penicillium herquei* CMB-MF025, *Paecilomyces* sp. CMB-MF010 (viridicatumtoxins) [10], and *Penicillium citrinum* CMB-MF006; none produced **5**.

## 3. Discussion

### 3.1. Origins of Prolinimines **1**–**4**, N-Amino-l-Proline Methyl Ester (**5**) and Furans **7**–**8**

Based on our observations we conclude that prolinimines A–D (**1**–**4**) are artifacts, while *N*-amino-l-proline methyl ester (**5**) is a natural product, and the furans **7–8** are media constituents. The rationale for these relationships is articulated below (i–viii);

(i) As the furans **7**–**8** are generated in the rice grain media during autoclaving, prior to fungal inoculation, they are best designated as media constituents.
(ii) CMB-F563 cultivations that produce the cryptic natural product *N*-amino-l-proline methyl ester (**5**), retain it exclusively within fungal mycelia.
(iii) The rupture of CMB-F563 mycelia during solvent extraction allow **5** to come in contact with media constituent furans **7**–**8**, facilitating rapid in situ formation of the Schiff base prolinimines A–B (**1**–**2**). As **1–2** are produced as a direct result of the extraction process they are more aptly characterized as artifacts.
(iv) During fractionation/handling prolinimine A (**1**) undergoes acid-mediated conversion to prolinimines C–D (**3**–**4**), consistent with **3**–**4** being artifacts.
(v) Autoclaving media with low carbohydrate content (i.e., M1) fails to produce significant levels of the furans **7**–**8**.
(vi) Those CMB-F563 cultivations that produce the natural product **5**, but lack the media components **7**–**8** (i.e., M1), are incapable of forming the artifacts **1**–**4**.
(vii) As **5** has cryptic physical and spectroscopic properties that make it hard to detect in cultures/extracts, it easily overlooked (unless you know what you are looking for, and apply appropriate measures—i.e., 2,4-DNB derivatization).
(viii) Notwithstanding, as **5** can be readily detected in culture/extracts by in situ chemical derivatization with 2,4-DNB to form the Schiff base **9**, it can be designated as a natural product.

### 3.2. Knowledge of N-Amino-Prolines in Nature

**Figure 10:**
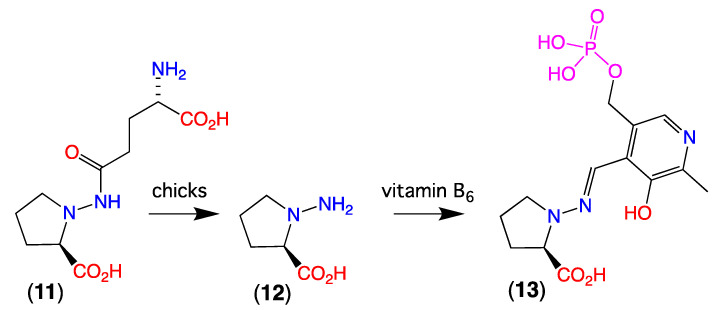
Flaxseed linatine (**11**), hydrolysis product *N*-amino-d-proline (**12**), and vitamin B_6_ Schiff base **13.**

Reports on natural products featuring the *N*-amino-proline moiety are extremely rare, with the only known example being the flaxseed dipeptide linatine (**11**) [11,12,13]. Although flaxseed meal is rich in protein, and a potential food source, when ingested by chicks **11** undergoes hydrolysis to *N*-amino-d-proline (**12**), which in turn forms an irreversible Schiff base (**13**) with vitamin B6, leading to a lethal vitamin B6 deficiency syndrome (Figure 10)—rendering flaxseed meal unsuitable for feeding chicks. 

Interestingly, whereas flaxseed *N*-amino-d-proline (**12**) is protected from premature Schiff base formation with biological important aldehydes as the amide pro-drug linatine (**11**), CMB-F563 retains *N*-amino-l-proline methyl ester (**5**) within mycelia to achieve a comparable level of control. This raises a number of interesting questions.

- Is flaxseed the source of linatine (**12**), or is **12** a fungal natural product? If the latter can flaxseed meal be treated to render it suitable as a chick feed?
- What is the ecological (survival) advantage accrued to CMB-F563 by its ability to produce **5**, and is there any correlation between this and the isolation of CMB-F563 from a fish gastrointestinal tract?
- Given the cryptic nature of **5**, how common is it (or other *N*-amino amino acids) as a fungal natural product?

## 4. Materials and Methods

### 4.1. General Experimental Details

Chiroptical measurements ([α]_D_) were obtained on a JASCO P-1010 polarimeter (JASCO International Co. Ltd., Tokyo, Japan) in a 100 × 2 mm cell at 20.5 °C. NMR spectra were obtained on a Bruker Advance DRX600 spectrometer (Bruker Pty. Ltd., Alexandria, Australia), in the solvents indicated and referenced to residual signals (δ_H_ 3.31 and δ_C_ 49.15 for MeOH) in deuterated solvents. Electrospray ionization mass spectra (ESIMS) were acquired using Agilent 1100 Series separations module equipped with an Agilent 1100 Series LC/MS mass detector (Agilent Technologies Inc., Mulgrave, Australia) in both positive and negative ion modes under the following conditions (Zorbax C_8_ 5 μm column, 150 × 4.6 mm, eluting with 1.0 mL/min 90% H_2_O/MeCN to 100% MeCN (with isocratic 0.05% HCO_2_H modifier) over 15 min, at 210 and 254 nm). Ultra high-performance liquid chromatograms (UHPLC) were obtained on Agilent 1290 infinity UHPLC system (Agilent Technologies Inc., Mulgrave, Australia) composed of 1290 infinity quaternary pump, thermostat, autosampler and diode array detector (Zorbax C_8_ RRHD 1.8 μm column, 50 × 2.1 mm, eluting with 0.417 mL/min 90% H_2_O/MeCN to 100% MeCN (with isocratic 0.01% TFA modifier) over 2.50 min, with detection at 210, 254, 300, and 400 nm). High-resolution ESIMS measurements were obtained on a Bruker micrOTOF mass spectrometer (Bruker Daltonic Pty. Ltd., Preston, Australia) by direct infusion in MeCN at 3 μL/min using sodium formate clusters as an internal calibrant. HPLC was performed using an Agilent 1100 Series diode array and/or multiple wavelength detectors and Agilent 1100 Series fraction collector (Agilent Technologies Inc., Mulgrave, Australia). Chemicals 5-hydroxymethyfurfural (**7**), 2,5-furandicarboxaldehyde (**8**) and 2,4-dinitrobenzaldehyde (2,4-DNB) were purchased from Sigma-Aldrich.

### 4.2. Collection and Taxonomy of *Evlachovaea* sp. CMB-F563

The fungus CMB-F563 was isolated from the gastrointestinal tract of a specimen of *Mugil* mullet fish, on M1 agar that consists of peptone (2.0 g), yeast extract (4.0 g), and starch (10.0 g), agar (18.0 g) and distilled water (1 L) at pH 7.0. The plates were incubated at 30 °C for 7 days. Genomic DNA was extracted from the mycelia using the DNeasy Plant Mini Kit (Qiagen) as per the manufacturer’s protocol. The 18s rRNA genes were amplified by PCR using the universal primers ITS 1 (5”-TCCGTAGGTGAACCTGCGG-3”) and ITS 4 (5”TCCTCCGCTTATTGATATGC-3”) purchased from Sigma-Aldrich. The PCR mixture (50 μL) contained genomic DNA (2 μL, 20–40 ng), EmeraldAmpn GT PCR Master Mix (2× Premix) (25 μL), primer (0.2 μM, each), and H_2_O (up to 50 μL). PCR was performed using the following conditions: initial denaturation at 95 °C for 2 min, 40 cycles in series of 95 °C for 20 s (denaturation), 56 °C for 20 s (annealing), and 72 °C for 30 s (extension), followed by one cycle at 72 °C for 5 min. The PCR products were purified with PCR purification kit (Qiagen) and sequenced. BLAST analysis (NCBI database) showed that the amplified ITS sequence has the closest homology with other members of the genus *Evlachovaea*. (Note: the earlier report on prolinimines from CMB-F563 assigned this strain to the genus *Trichoderma*). The previous *Tricoderma* assignment was determined using RefSeq nucleotide sequences while generating the phylogenetic tree. Careful morphological examination revealed that the fungus does not belong to the *Tricoderma* genus. Therefore, the phylogenetic tree was repeated using Genbank nucleotide sequences rather than RefSeq nucleotide sequences to reveal that CMB-F563 belongs to *Evlachovaea* (Figures S1 and S2 in Supporting Information). Accession number: MG561929. 18S rRNA sequence:

GATTCGAGGTCAACGTTCAGAAGTTGGGTGTTTTACGGCGTGGCCACGTCGGGGTTCCGGTGCGCGTTGAGTTACTACGCAGAGGTCGCCGCGGACGGGCCGCCACTTCATTTCAGGGGCGGCGGGGTAGTGCCGTCCCCCAACGCCGACCCCACTAAGCGCGGGGTCGAGGGGTGAAATGACGCTCGAACAGGCATGCCCGCCAGAATGCTGGCGGGCGCAATGTGCGTTCAAAGATTCGATGATTCACTGAATTCTGCAATTCACATTACTTATCGCATTTCGCTGCGTTCTTCATCGATGCCAGAACCAAGAGATCCGTTGTTGAAAGTTTTGATTCATTTGTTTTGCCTTGCGGCGGATTCAGAAGACGTAAAGAATACAGAGTTTGGGGTCCCCGGCGGCCGCCTGGGTCCGGGTCGCGGGCGGCGCAGGGCCGTCCGGACGCCGGGGCGGGTCCGCCGAAGCAACGATTGGTATGTTCACATAGGGTTGGGAGTTGAAAACTCGTTAATGATCCCTCCGCAGGTTCCCCTACGGAGG

### 4.3. Synthesis of Authentic Standards

#### 4.3.1. Synthesis of *N*-Amino-l-Proline Methyl Ester (**5**) 

A sample of l-proline (1 g, 8.69 mmol) and NaNO_2_ (6 g) in water (10 mL) was treated with H_2_SO_4_ (3 M, 16 mL) at room temperature over 10 min, after which the reaction mixture was extracted with diethyl ether (4 × 25 mL). The combined organic layers were concentrated in vacuo to yield a 1.8:1 mixture of the *syn* and *anti* isomers of *N*-nitroso-l-proline (865 mg, 69%) as a white solid. ^1^H NMR (600 MHz, methanol-*d*_4_),(mixture of isomers, syn: anti = 1.8:1) *δ* 5.28 (dd, J = 8.6, 3.1 Hz, 1H), 4.49 (dd, J = 8.6, 5.0 Hz, 1.8H), 4.46–4.42 (m, 1.8H), 4.35–4.30 (m, 1.8H), 3.70–3.60 (m, 2H), 2.47–2.36 (m, 2.8H), 2.33–2.28 (m, 1H), 2.23–2.16 (m, 1.8H), 2.12–1.99 (m, 5.6H); ^13^C NMR (150 MHz, MeOH-*d*_4_) *δ*_c_ 174.0, 172.3, 63.7, 59.9, 51.5, 47.1, 30.0, 29.1, 24.3, 22.2; HRESI(+)MS *m*/*z* 167.0432 [M + Na]^+^ (calcd for C_5_H_8_N_2_NaO_3_ 167.0427).

A sample of *N*-nitroso-l-proline (1.2 g, 8.3 mmol) dissolved in anhydrous MeOH (40 mL) and conc. H_2_SO_4_ (700 μL) was stirred at room temperature overnight after which the reaction mixture was concentrated in vacuo and the residue dissolved in DCM (40 mL) and washed with saturated aqueous NaHCO_3_ (40 mL). After decanting the DCM layer, the aqueous layer was re-extracted with DCM (3 × 40 mL) and the combined DCM extracts were dried over Na_2_SO_4_, filtered and concentrated in vacuo to yield 1.6:1 mixture of the *syn* and *anti* isomers of *N*-nitroso-l-proline methyl ester (1.03 g, 78%) as a yellow oil. ^1^H NMR (600 MHz, CDCl_3_), (mixture of isomers, syn: anti = 1.6:1), *δ* 5.23 (dd, J = 8.5, 3.2 Hz, 1H), 4.45 (dd, J = 8.0, 4.6 Hz, 1.6H), 4.42–4.38 (m, 1.6H), 4.35–4.30 (m, 1.6H), 3.74 (s, 3H), 3.66 (s, 4.8H), 3.67–3.56 (m, 2H), 2.36–2.23 (m, 4.6H), 2.20–2.14 (m, 1.6H), 2.06–1.97 (m, 5.2H); ^13^C NMR (150 MHz, MeOH-*d*_4_) *δ*_c_ 171.0, 169.2, 62.0, 58.0, 52.9, 52.6, 50.0, 45.7, 29.0, 27.8, 23.3, 21.2; HRESI(+)MS *m*/*z* 181.0591 [M + Na]^+^ (calcd for C_6_H_10_N_2_NaO_3_ 181.0584).

Aliquots of *N*-nitroso-l-proline methyl ester (250–363 mg, 1.58–2.29 mmol) were reduced with Zn dust (1.0–1.4 g) in 50% AcOH (12.5–18 mL) at 0 °C over 15 min, and HPLC-MS analysis confirmed quantitative conversion to *N*-amino-l-proline methyl ester (**5**); HRESI(+)MS *m*/*z* 145.0973 [M + H]^+^ (calcd for C_6_H_13_N_2_O_2_ 145.0972).

#### 4.3.2. Synthesis of 2,4-Dinitrobenzaldehyde Schiff Base 9

An aliquot of **5** (20 mg, 0.14 mmol) dissolved in anhydrous MeOH (5 mL) was treated with 2,4-dinitrobenzaldehyde (20 mg, 0.10 mmol) at room temperature for 4 h, after which the reaction mixture was quenched with water (5 mL), extracted with EtOAc (2 × 5 mL), concentrated in vacuo, and purified by preparative reversed phase HPLC (Phenomenex Luna-C_18_, 21.2 mm × 25 cm, 10 μm, 20 mL/min, gradient elution 50% H_2_O/MeCN to MeCN over 15 min) to yield **9** (15 mg, 46.6% yield): [α]^22^_D_ –170.6 (*c* 0.01, MeOH); 1D and 2D NMR (600 MHz, methanol-*d*_4_) see Table S1 and Figures S3–S5 in Supporting Information; HRESI(+)MS *m*/*z* 345.0819 [M + Na]^+^ (calcd for C_13_H_14_N_4_NaO_6_ 345.0806).

#### 4.3.3. Synthesis of 2,4-Dinitrobenzaldehyde Schiff Base 10

An aliquot of *N*-nitroso-l-proline (50 mg, 0.34 mmol) was reduced with Zn dust (0.5 g) in 50% AcOH (10 mL) at 0 °C for 15 min, to yield a solution containing *N*-amino-l-proline (**6**), which was filtered and dried in vacuo without further purification. An aliquot of **6** (45 mg, 0.34 mmol) dissolved in anhydrous MeOH (5 mL) was treated with 2,4-dinitrobenzaldehyde (59 mg, 0.30 mmol) at room temperature for 4 h, after which the reaction mixture was quenched with water (5 mL), extracted with EtOAc (2 x 5 mL), and concentrated in vacuo, to yield crude **10** (90 mg, 97%). A portion (12.7 mg) was purified by preparative reversed phase HPLC (Phenomenex Luna-C_18_, 21.2 mm × 25 cm, 10 μm, 20 mL/min, gradient elution 90% H_2_O/MeCN to MeCN over 30 min) to yield **10** (9.5 mg, 74.8%): [α]^22^_D_ –205.6 (*c* 0.01, MeOH); 1D and 2D NMR (600 MHz, methanol-*d*_4_) see Table S2 and Figures S6–S8 in Supporting Information; HRESI(+)MS *m*/*z* 331.0645 [M + Na]^+^ (calcd for C_12_H_12_N_4_NaO_6_ 331.0649).

### 4.4. The Presence of Furans **7**–**8**, and Absence of **5**, in Uninoculated Rice Grain Media

Matured (30 d) uninoculated rice grain media (25 g) that consists of medium grain rice (700g), peptone (3.0 g), yeast extract (3.0 g), monosodium glutamate (1.0 g), and distilled water (1 L). The rice medium was extracted with EtOAc (50 mL), with and without coaddition of *N*-methyl-l-proline methyl ester (**5**) (20 mg in 5 mL MeOH). Analytical samples (1 mL) were removed from the mixture at regular intervals over 24 h, and immediately dried under N_2_ at 40 °C. Analytical samples were suspended in MeOH (40 μL), filtered (0.45 μm), and subjected to HPLC-DAD-ESIMS analysis with single ion extraction (SIE).

### 4.5. Detection of N-Aminoprolines **5**–**6** Using 2,4-Dinitrobenzaldehyde

Samples of uninoculated M1 broth (1.5 mL) in a 24-well microbioreactor format were treated with an aliquot (40 μL) of stock solutions (5 mg/mL in H_2_O) of either *N*-amino-l-proline methyl ester (**5**) or its free acid **6**. Both solutions were also treated with an aliquot (40 μL) of 2,4-DNB (5 mg/mL in 20% aqueous DMSO) and extracted with EtOAc (2 mL) in situ at 150 rpm for 60 min. The organic layer was filtered (0.45 μm), dried under N_2_ at 40 °C, and the resulting extracts redissolved in MeOH (200 μL) and subjected to UHPLC-DAD analysis.

### 4.6. Detection of N-Amino-l-Proline Methyl Ester (**5**) in CMB-F563 Rice Grain Media Cultivations

Matured (30 d) rice media (25 g) cultivations of CMB-F563 were extracted with EtOAc (200 mL) by shaking at 150 rpm with and without coaddition of 2,4-DNB (60 mg in 12 mL of 20% aqueous DMSO). Analytical samples (1 mL) were removed at regular intervals over 48 h and immediately dried under N_2_ at 40 °C. Analytical samples were then suspended in MeOH (40 μL), filtered (0.45 μm), and subjected to HPLC-DAD-ESIMS analysis with single ion extraction (SIE).

### 4.7. Detection of N-Amino-l-Proline Methyl Ester (**5**) in Uninoculated M1 Broth Media 

Samples of uninoculated M1 broth (1.5 mL) in a 24-well microbioreactor format were extracted with EtOAc (2 mL) in situ at 150 rpm for 60 min with and without coaddition of an aliquot (40 μL) of *N*-amino-l-proline methyl ester (**5**) (5 mg/mL in H_2_O). The organic layer was filtered (0.45 μm), dried under N_2_ at 40 °C, and the resulting residues redissolved in MeOH (40 μL) and subjected to UHPLC-DAD analysis.

### 4.8. Detection of N-Amino-l-Proline Methyl Ester (**5**) in CMB-F563 M1 Broth Media Cultivations

Two M1 static broth (1.5 mL) cultivations of CMB-F563 were incubated at 26.5 °C for 9 days in a 24-well microbioreactor format. Each culture was treated with an aliquot (40 μL) of 2,4-DNB (5 mg/mL in 20% aqueous DMSO) and extracted with EtOAc (2 mL) in situ at 150 rpm for 60 min. The organic layers were filtered (0.45 μm), dried under N_2_ at 40 °C, and the resulting residues dissolved in MeOH (40 μL) and subjected to UHPLC-DAD analysis.

### 4.9. Mycelial Secretion Status of N-Amino-l-Proline Methyl Ester (**5**)

#### 4.9.1. Using 2,4-Dinitrobenzaldehyde (2,4-DNB) as the Derivatizing Agent

An M1 static broth (5 mL) cultivation of CMB-F563 was incubated at 26.5 °C for 9 days after which the mycelia were physically separated from the culture broth using an inoculation loop. The mycelia and culture broth were then individually extracted with EtOAc (5 mL) by shaking at 150 rpm for 60 min with coaddition of an aliquot (150 μL) of 2,4-DNB (5 mg/mL in 20% aqueous DMSO). The organic layers were filtered (0.45 μm), dried under N_2_ at 40°C, and the residues redissolved in MeOH (120 μL) and subjected to UHPLC-DAD analysis.

#### 4.9.2. Using 5-Hydroxymethyfurfural (**7**) as the Derivatizing Agent

An M1 static broth (5 mL) cultivation of CMB-F563 was incubated at 26.5 °C for 9 days after which the mycelia were physically separated from the culture broth using an inoculation loop. The mycelia and culture broth were then individually extracted with EtOAc (5 mL) by shaking at 150 rpm for 60 min with coaddition of an aliquot (150 μL) of 5-hydroxymethyfurfural (**7**) (5 mg/mL in water). The organic layers were filtered (0.45 μm), dried under N_2_ at 40°C, and the residues redissolved in MeOH (120 μL) and subjected to UHPLC-DAD analysis.

### 4.10. Detection of N-Amino-l-Proline Methyl Ester (**5**) in Other CMB-F563 Broth Media Cultivations

A panel of 11 broth media (MATRIX) inoculated with an aliquot (20 μL) of CMB-F563 seed culture and incubated for 9 days at 26.5 °C, were individually extracted with EtOAc (2 mL) in situ at 150 rpm for 60 min, with and without coaddition of an aliquot (40 μL) of 2,4-DNB (5 mg/mL in 20% aqueous DMSO). The organic layers were filtered (0.45 μm), dried under N_2_ at 40 °C, and the resulting residues dissolved in MeOH (40 μL) and subjected to UHPLC-DAD analysis.

### 4.11. Detection of N-Amino-l-Proline Methyl Ester (**5**) in Different Fungal Genera

Nine fungi chosen from Capon Lab collection (*Fusarium* sp. CMB-NF041, *Aspergillus terreus* CMB-M0231F, *Penicillium roseopurpureum* CMB-MF038, *Eupenicillium javanicum* CMB-MF036, *Chaunopycnis* sp. CMB-MF028, *Beauvaria bassiana* CMB-MF026, *Penicillium herquei* CMB-MF025, *Paecilomyces* sp. CMB-MF010, and *Penicillium citrinum* CMB-MF006) in PYG static broth (1.5 mL) in a 24-well microbioreactor format, were incubated at 26.5 °C for 9 days. The cultures were treated with an aliquot (40 μL) of 2,4-DNB (5 mg/mL in 20% aqueous DMSO) and extracted with EtOAc (2 mL) in situ at 150 rpm for 60 min. The organic layers were filtered (0.45 μm), dried under N_2_ at 40 °C, and the resulting residues redissolved in MeOH (40 μL) and subjected to UHPLC-DAD analysis. 

## 5. Conclusions

Our further investigations into the chemistry of *Evlachovaea* sp. CMB-F563 returned unexpected results, including the discovery of a new cryptic natural product, *N*-amino-l-proline methyl ester (**5**), only the second reported natural occurrence of the *N*-aminoproline motif, and the first of microbial origin. To enable this discovery, we developed a high sensitivity in situ 2,4-DNB analytical derivatization methodology, capable of detecting low levels of **5** in culture extracts. This methodology proved highly effective, and we are currently applying it to detect other known/unknown cryptic biogenic amines. 

Our investigations also drew attention to the largely overlooked but critical role played by culture media components (i.e., furans **7**–**8**), as well as the phenomena whereby fungi protect the integrity of highly reactive natural products (i.e., **5**) by sequestering them within mycelia. We also demonstrate that although prolinimines A–B (**1**–**2**) can be detected in fresh cultivation extracts, they are nevertheless artifacts of the solvent extraction process.

Finally, our experience with CMB-F563 challenged our perception of the distinction between a microbial natural product, an artifact, a media component, and a contaminant, as encapsulated in the following observations.

**A natural product** is a chemical that originates from, and can be detected in a fresh unfractionated extract of, a source organism, provided the process of extraction and/or detection does not initiate a chemical transformation that is solely responsible for producing the chemical.

**An artifact** is a natural product that has undergone a chemical transformation during extraction, handling, storage and/or analysis. 

**A media component** is a chemical that is present in the autoclaved culture media prior to microbial inoculation. 

**A contaminant** is a chemical that is detected in and/or isolated from a natural extract, but is neither a natural product, or an artifact, or a media component.

Significantly, some artifacts (even media components and contaminants) are not immediately recognised as such, and have been and continue to be misidentified in the scientific literature as natural products.

## Data Availability

Data is contained within the article or Supplementary Material.

## Supplementary Materials

The following are available online at https://www.mdpi.com/1660-3397/19/3/151/s1, phylogenetic tree, tabulated 1D and 2D NMR data, annotated 1D NMR and selected 2D NMR spectra and HPLC chromatograms.
