# Supplementary material for: N-Amino-l-Proline Methyl Ester from an Australian Fish Gut-Derived Fungus: Challenging the Distinction between Natural Product and Artifact
Source: Mar Drugs. 2021 Mar 12;19(3):151. doi: 10.3390/md19030151 (PMC7999761; doi:10.3390/md19030151)
Supplement: Supplementary file 1 [file marinedrugs-19-00151-s001.pdf]

N-Amino-L-proline methyl ester from an Australian fish gut fungus: Challenging the distinction between natural product and artifact.

Osama G. Mohamed,[1,2] Zeinab G. Khalil[1] and Robert J. Capon[1]

[1]Division of Chemistry and Structural Biology, Institute for Molecular Bioscience, The University of Queensland, St Lucia, QLD 4072, Australia

KEYWORDS (*N*-amino-L-proline methyl ester; prolinimines; 5-hydroxymethyfurfural; 2,5-furandicarboxaldehyde; Schiff base; artifact; media component)

[2] current address University of Michigan Life Sciences Institute, Natural Products Discovery Core, Ann Arbor 48109, Michigan, USA

Corresponding author.

* Tel +61 7 3346 2979. Facsimile +61 7 3346 2090. Email: r.capon@uq.edu.au

Table of Contents

Section 1 Collection and Taxonomy of *Evlachovaea* sp. CMB-F563 3

Section 2 NMR data of synthetic 2,4-dinitrobenzaldehyde Schiff bases (**9**) and (**10**) 5

List of Figures

Figure S1. Blast search (closest match) for CMB-F563 3

Figure S2. Phylogenetic analysis of CMB-F563 using PhyML Maximum likelihood analysis of ITS DNA sequences using the optimal nucleotide substitution model determined by jModeltest2 using UGENE software. Phylogenetic relationship of CMB-F563 with selected strains (nucleotide collection Genbank) shown with their accession numbers indicated in brackets, highlighted in blue. 4

Figure S3. ^{1}H NMR (600 MHz, methanol-d_4) spectrum for 2,4-dinitrobenzaldehyde Schiff base (**9**). * Residual methanol solvent 5

Figure S4. ^{13}C NMR (150 MHz, methanol-d_4) spectrum for 2,4-dinitrobenzaldehyde Schiff base (**9**) 5

Figure S5. 2D NMR ROESY (methanol-d_4) spectrum for 2,4-dinitrobenzaldehyde Schiff base (**9**) 6

Figure S6. ^{1}H NMR (600 MHz, methanol-d_4) spectrum for 2,4-dinitrobenzaldehyde Schiff base (**10**) 7

Figure S7. ^{13}C NMR (150 MHz, methanol-d_4) spectrum for 2,4-dinitrobenzaldehyde Schiff base (**10**) 7

Figure S8. 2D NMR ROESY (methanol-d_4) spectrum for 2,4-dinitrobenzaldehyde Schiff base (**10**) 8

Figure S9. UHPLC-DAD (400 nm) chromatograms of synthetic **9** and **10** and their UV-vis (UHPLC-DAD, H_2O/MeCN plus TFA) spectrum (inset).. 9

List of Tables

Table S1. 1D and 2D NMR (600 MHz, methanol-d_4) data for 2,4-dinitrobenzaldehyde Schiff base (**9**) 6

Table S2. 1D and 2D NMR (600 MHz, methanol-d_4) data for 2,4-dinitrobenzaldehyde Schiff base (**10**) 8

Section 1 Collection and Taxonomy of *Evlachovaea* sp. CMB-F563

BLAST search (closet match)

An NCBI-BLAST database search of 18s rRNA sequence established a 99% similarity for CMB-F563 with *Evlachovaea* sp. (Figure S1. Blast search (closest match) for CMB-F563

☐	Evlachovaea sp. 126 18S ribosomal RNA gene, partial sequence; internal transcribed spacer 1, 5.	948	948	96%	0.0	99%	EU553279.1
☐	Evlachovaea sp. IP 304 internal transcribed spacer 1, partial sequence; 5.8S ribosomal RNA gene	915	915	92%	0.0	99%	GU734765.1
☐	Cordyceps sp. BCMU CN03 genes for 18S ribosomal RNA, internal transcribed spacer 1, 5.8S rib	981	981	100%	0.0	99%	AB233335.1
☐	Hypocreales sp. NWHC 24746-01-01-02 18S ribosomal RNA gene, partial sequence; internal tran	876	876	89%	0.0	99%	KX148701.1
☐	Fungal sp. GFI 32 5.8S rRNA gene, 28S rRNA gene (partial), ITS1 and ITS2, isolate GFI 32	957	957	98%	0.0	99%	AJ609125.1
☐	Isaria fumosorosea strain ARSEF 1576 18S ribosomal RNA gene, partial sequence; internal trans	909	909	93%	0.0	99%	EU553296.1
☐	Evlachovaea sp. AB-2016 18S ribosomal RNA gene, partial sequence; internal transcribed spacer	830	830	86%	0.0	99%	KU983517.1
☐	Verticillium sp. IMI 331591R internal transcribed spacer 1, 5.8 S ribosomal RNA gene, and interna	815	815	88%	0.0	97%	EF513024.1
☐	Cordyceps pruinosa voucher HMIGD 20930 18S ribosomal RNA gene, partial sequence; internal t	787	787	88%	0.0	96%	DQ342253.1
☐	Cordyceps pruinosa gene for 18S rRNA, ITS1, 5.8S rRNA, ITS2, 26S rRNA, partial and complete :	880	880	99%	0.0	96%	AB044635.1
☐	Simplicillium sp. AMVP-2014 18S ribosomal RNA gene, partial sequence; internal transcribed spa	848	848	98%	0.0	96%	KF937365.1
☐	Ophiocordyceps caloceroides voucher TSJ871 18S ribosomal RNA gene, partial sequence; intern	800	800	96%	0.0	95%	KF937319.1
☐	Isaria fumosorosea isolate IfTS02 internal transcribed spacer 1, partial sequence; 5.8S ribosomal	782	782	97%	0.0	94%	KX057373.1
☐	Paecilomyces sp. (in: Hypocreales) strain CICR-RSS-0089 small subunit ribosomal RNA gene, pa	780	780	98%	0.0	93%	MG976232.1
☐	Isaria javanica voucher CHE-CNRCB 307/7 18S ribosomal RNA gene, partial sequence; internal t	780	780	98%	0.0	93%	KM234218.1
☐	Isaria javanica voucher CHE-CNRCB 303/2 18S ribosomal RNA gene, partial sequence; internal t	780	780	98%	0.0	93%	KM234213.1
☐	Akanthomyces websteri strain BCC02113 18S ribosomal RNA gene, partial sequence; internal tra	780	780	98%	0.0	93%	GQ250008.1
☐	Paecilomyces sp. ZLY-2010 isolate M26 internal transcribed spacer 1, partial sequence; 5.8S ribos	780	780	98%	0.0	93%	HM595502.1
☐	Isaria javanica voucher CHE-CNRCB 305/1 18S ribosomal RNA gene, partial sequence; internal t	774	774	97%	0.0	93%	KM234215.1
☐	Isaria javanica voucher CHE-CNRCB 305 18S ribosomal RNA gene, partial sequence; internal tra	773	773	97%	0.0	93%	KM234214.1
☐	Isaria javanica voucher CHE-CNRCB 303 18S ribosomal RNA gene, partial sequence; internal tra	773	773	97%	0.0	93%	KM234212.1
☐	Isaria javanica strain RCEF5499 18S ribosomal RNA gene, partial sequence; internal transcribed :	771	771	97%	0.0	93%	KP644424.1
☐	Isaria javanica strain RCEF5495 18S ribosomal RNA gene, partial sequence; internal transcribed :	771	771	97%	0.0	93%	KP644423.1
☐	Isaria javanica voucher CHE-CNRCB 307 18S ribosomal RNA gene, partial sequence; internal tra	771	771	97%	0.0	93%	KM234217.1
☐	Isaria fumosorosea isolate NBAII Pfu-8 internal transcribed spacer 1, partial sequence; 5.8S ribos	771	771	97%	0.0	93%	KC147667.1
☐	Isaria javanica strain CM1 18S ribosomal RNA gene, partial sequence; internal transcribed spacer	771	771	97%	0.0	93%	EF990131.1
☐	Isaria javanica isolate ACP small subunit ribosomal RNA gene, partial sequence; internal transcrib	793	793	100%	0.0	93%	MG837718.1
☐	Isaria fumosorosea isolate FAFU-1 small subunit ribosomal RNA gene, partial sequence; internal t	793	793	100%	0.0	93%	MG837716.1
☐	Isaria fumosorosea isolate SKCH-1 18S ribosomal RNA gene, partial sequence; internal transcribe	793	793	100%	0.0	93%	FJ765017.1
☐	Isaria fumosorosea isolate NLAG 18S ribosomal RNA gene, partial sequence; internal transcribed	793	793	100%	0.0	93%	FJ765016.1

Figure S1. Blast search (closest match) for CMB-F563

Phylogenetic Tree and Microscopic image of CMB-F563

Phylogenetic tree obtained by PhyML Maximum Likelihood analysis was constructed using the top similar 18S rRNA sequences displayed after BLAST on Genbank RNA NCBI database using CMB-F563 18S rRNA as queries. The JC69 model was used to infer phylogeny

sequences.[1] Sequences alignments were produced with the MUSCLE program.[2] Phylogenetic tree was constructed using the UGENE program using the aforementioned models and visualized using Ugene's tree view (Figure S2). Based on the paper published by Humber et al[3], the morphology and the molecular taxonomy of *Evlachovaea* genus places this genus closely to *Isaria* and *Cordyceps* genera. In addition to the 18s rRNA sequence analysis, the taxonomic identification of CMB-F563 as a *Evlachovaea* sp. was supported by a microscopic examination of its conidia (i.e. fungal spores) and phialides (i.e. flask-shaped projections from the dilated part of the top of fungal conidia) (Figure S2 inset).

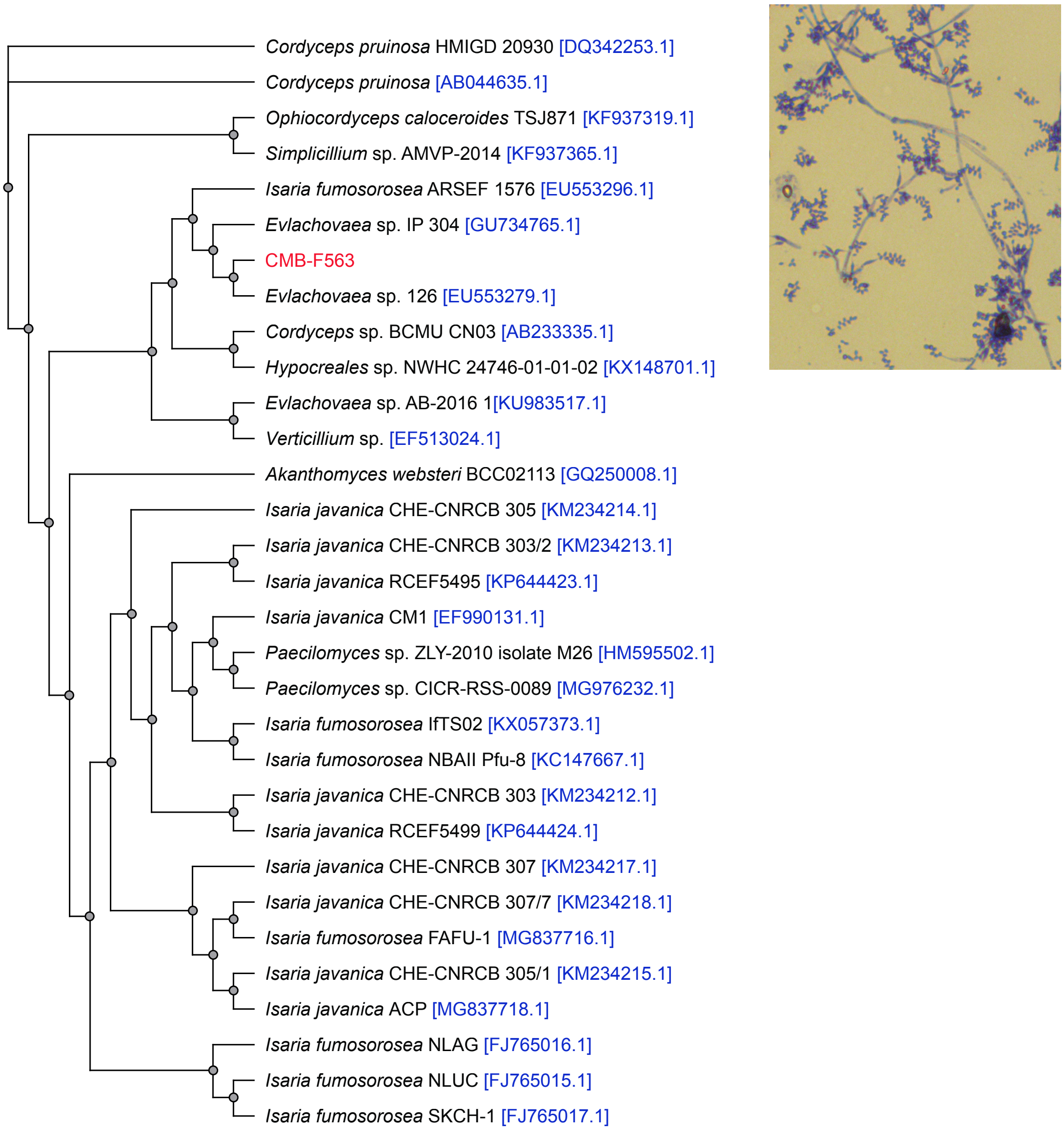

Figure S2. Phylogenetic analysis of CMB-F563 using PhyML Maximum likelihood analysis of ITS DNA sequences using the optimal nucleotide substitution model determined by jModeltest2[2] using UGENE software [2]. Phylogenetic relationship of CMB-F563 with selected strains (nucleotide collection Genbank) shown with their accession numbers indicated in brackets, highlighted in blue. Microscopic image for CMB-F563 (inset).

Section 2 NMR data of synthetic 2,4-dinitrobenzaldehyde Schiff bases (9) and (10)

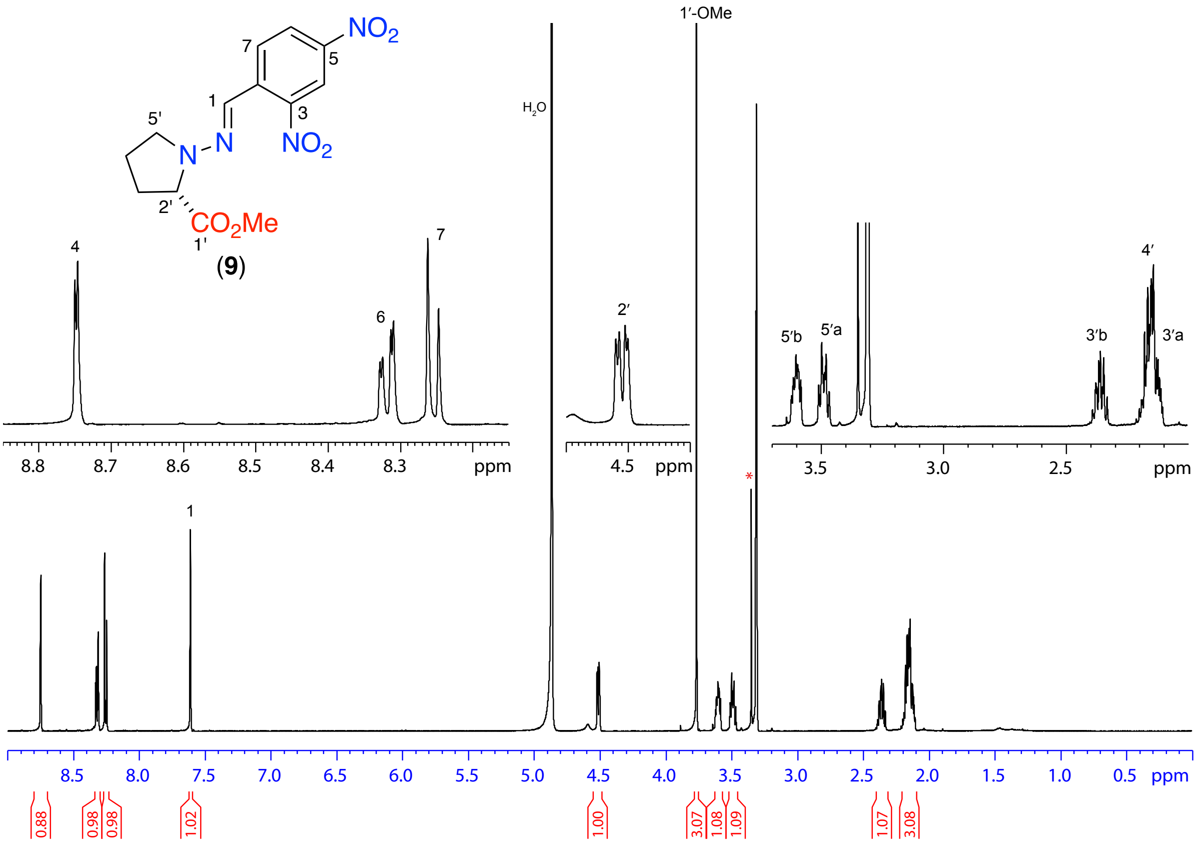

Figure S3. ^{1}H NMR (600 MHz, methanol-d_4) spectrum for 2,4-dinitrobenzaldehyde Schiff base (**9**). * Residual methanol solvent

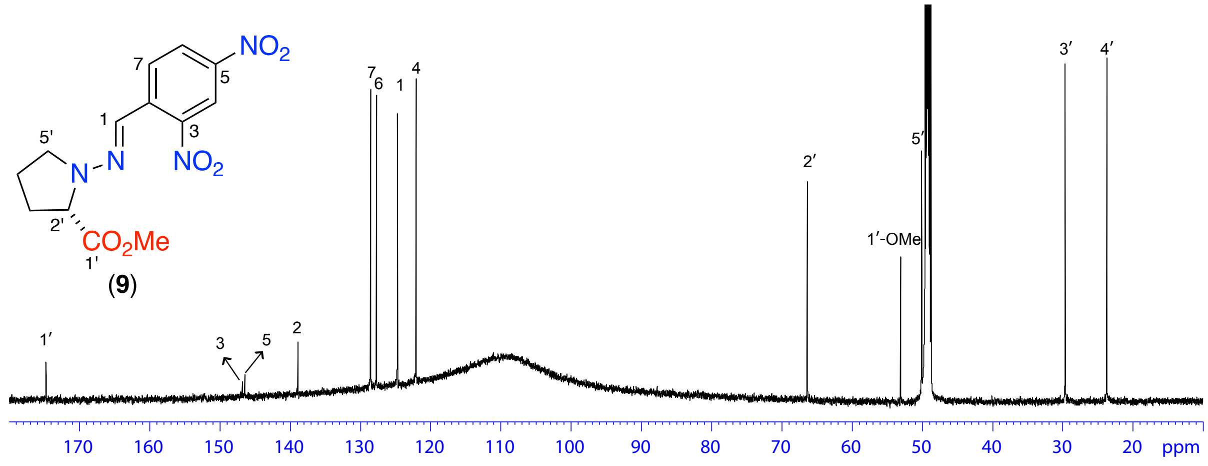

Figure S4. ^{13}C NMR (150 MHz, methanol-d_4) spectrum for 2,4-dinitrobenzaldehyde Schiff base (**9**)

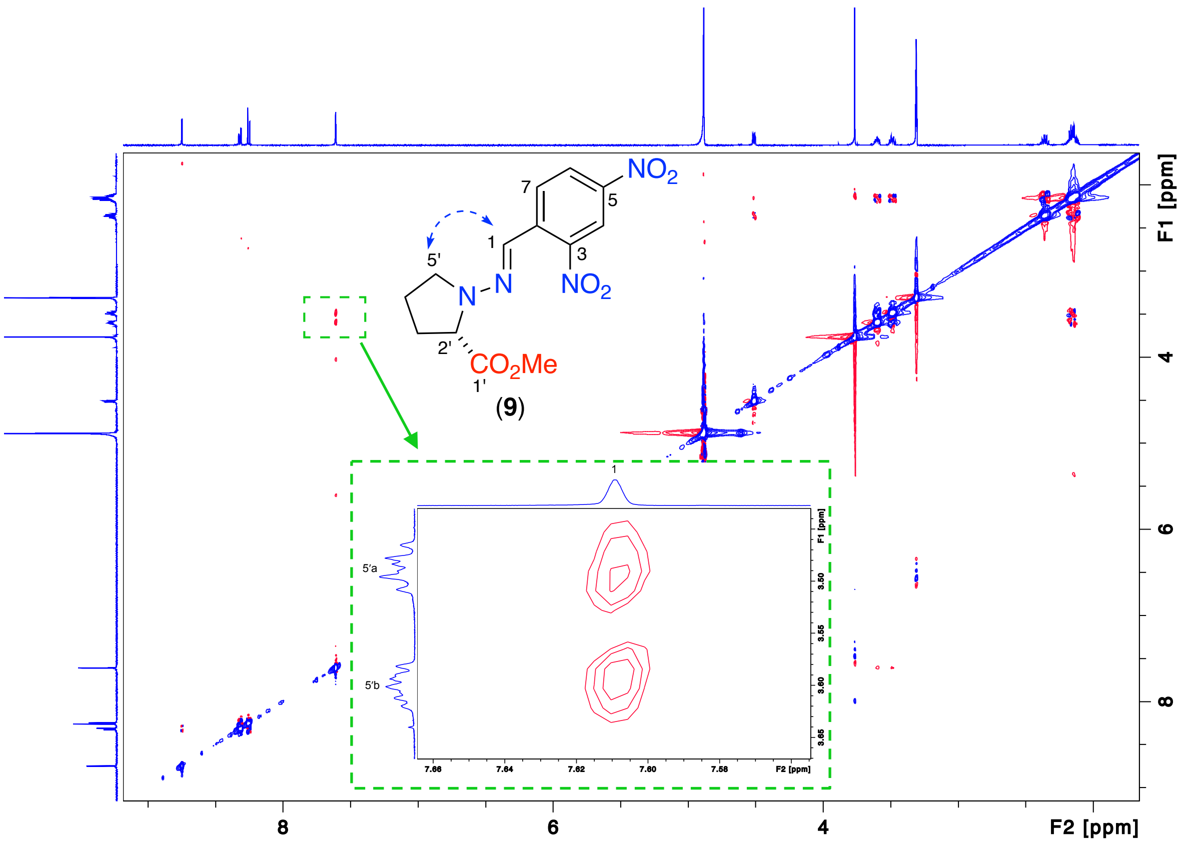

Figure S5. 2D NMR ROESY (methanol-d_4) spectrum for 2,4-dinitrobenzaldehyde Schiff base (**9**)

Table S1. 1D and 2D NMR (600 MHz, methanol-d_4) data for 2,4-dinitrobenzaldehyde Schiff base (**9**)

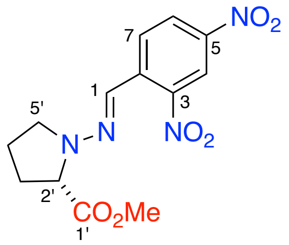

(**9**)

Pos.	δ_H, m (*J* Hz)	δ_C	*COSY*	*HMBC*	*ROESY*
1	7.61, s	124.7		2, 3, 7	5′a, 5′b
2	---	138.8			
3	---	146.7			
4	8.75, d (*2.2*)	122.0	6	2, 3, 5, 6	
5	---	146.4			
6	8.32, dd (*9.1, 2.2*)	127.7	4, 7	2, 4, 5	
7	8.25, d (*9.1*)	128.5	6	1, 3, 5	
1'	---	174.7			
2'	4.51, dd *(8.7, 3.0)*	66.3	3′a, 3′b	1′, 3′, 4′, 5′	
3'	a 2.16, m<br>b 2.36, m	29.6	2′, 3′b, 4′<br>2′, 3′a, 4′	1′, 2′, 4′	
4'	2.16, m	23.7	3′a, 3′b, 5′a, 5′b	2′, 3′, 5′	
5'	a 3.49, dd (*17.8, 7.4*)<br>b 3.60, m	50.0	4'<br>4'	2′, 3′, 4′	1<br>1
1'-OMe	3.77, s	53.0		1'	

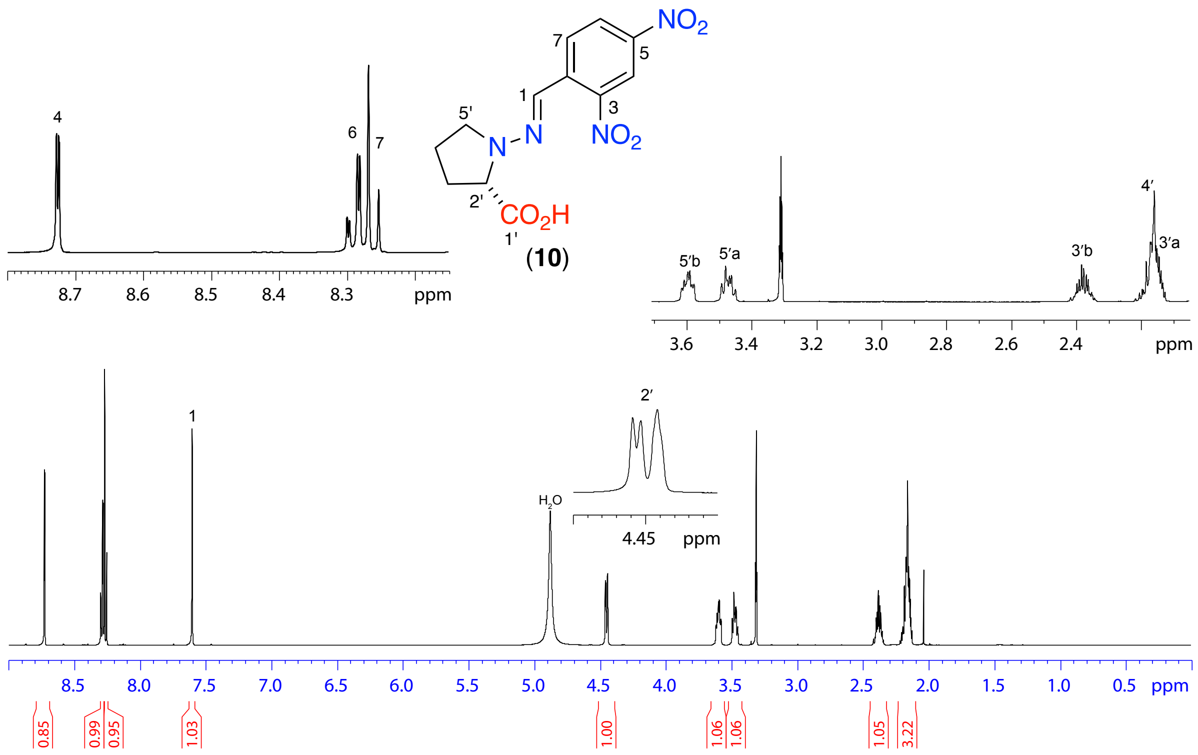

Figure S6. ^{1}H NMR (600 MHz, methanol-d_4) spectrum for 2,4-dinitrobenzaldehyde Schiff base (**10**)

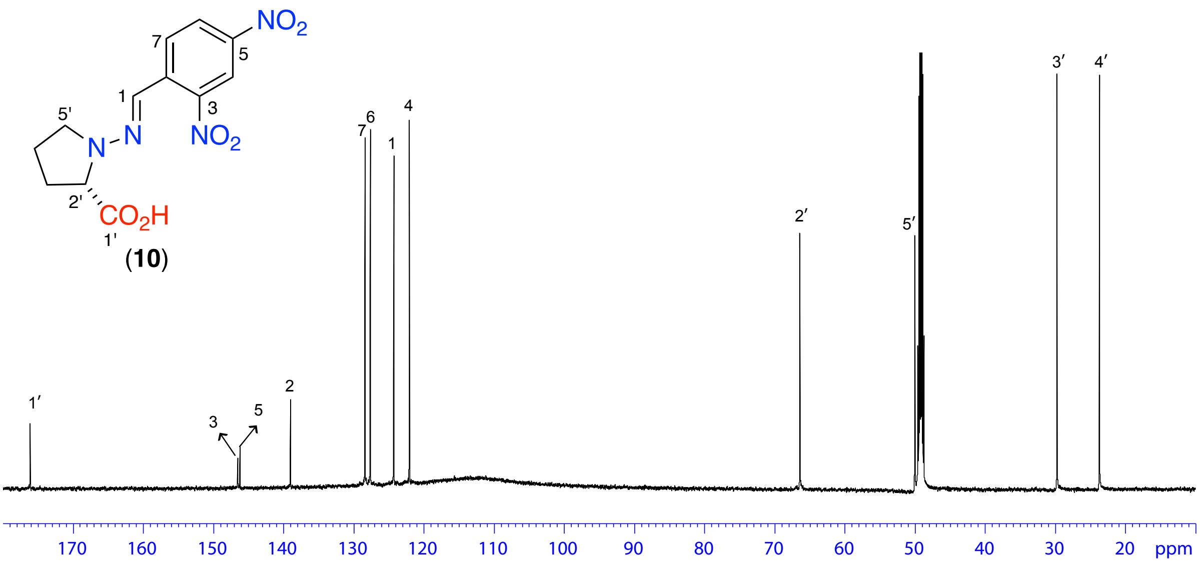

Figure S7. ^{13}C NMR (150 MHz, methanol-d_4) spectrum for 2,4-dinitrobenzaldehyde Schiff base (**10**)

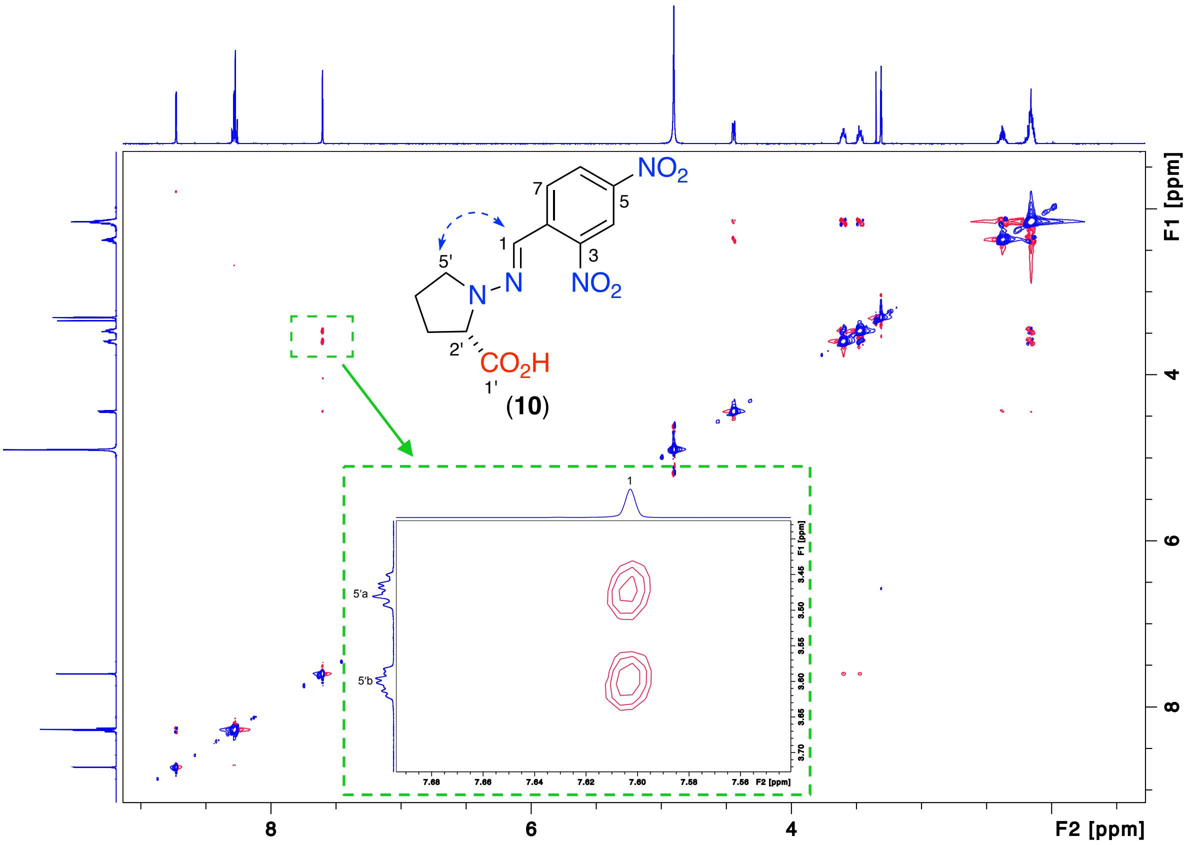

Figure S8. 2D NMR ROESY (methanol-d_4) spectrum for 2,4-dinitrobenzaldehyde Schiff base (**10**)

Table S2. 1D and 2D NMR (600 MHz, methanol-d_4) data for 2,4-dinitrobenzaldehyde Schiff base (**10**)

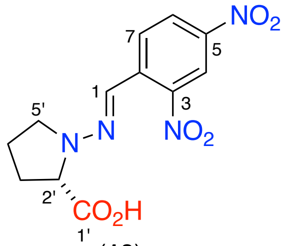

(**10**)

Pos.	δ_H, m (*J* Hz)	δ_C	*COSY*	*HMBC*	*ROESY*
1	7.60, s	124.2		2, 3, 7	2′, 5′a, 5′
2	---	138.9			
3	---	146.5			
4	8.72, dd (*2.2, 0.3*)	122.0	6	2, 3, 5, 6	
5	---	146.2			
6	8.29, ddd (*9.0, 2.2, 0.3*)	127.6	4, 7	2, 4, 5	
7	8.26, d (*9.0*)	128.4	6	1, 3, 5	
1'	---	176.1			
2'	4.45, dd *(8.7, 3.3)*	66.4	3′a, 3′b	1′, 3′, 4′, 5′	1
3'	a 2.15, m	29.8	2′, 3′b, 4′	1′, 2′, 4′	
	b 2.3, m		2′, 3′a, 4′		
4'	2.16, m	23.7	3′a, 3′b, 5′a, 5′b	2′, 3′, 5′	
5'	a 3.47, m	50.0	4'	2′, 3′, 4′	1
	b 3.60, m		4'		1

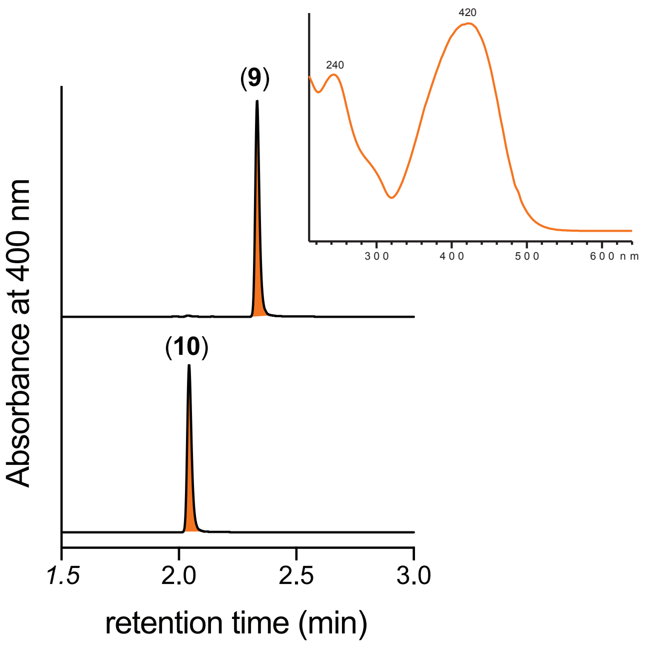

Figure S9. UHPLC-DAD (400 nm) chromatograms of synthetic **9** and **10** and their UV-vis (UHPLC-DAD, H_2O/MeCN plus TFA) spectrum (inset)

References:

1. Darriba, D.; Taboada, G. L.; Doallo, R.; Posada, D. *Nat. Methods* **2012**, 9(8), 772–772.

2. Okonechnikov, K.; Golosova, O.; Fursov, M.; UGENE team. *Bioinformatics* **2012**, 28(8), 1166–1167.

3. Humber, R.; Rocha, L.; Inglis, P.; Kipnis, A.; Luz, C. *Fungal Biology* **2013**, 117(1), 1-12.
